# Adiponectin levels and waist circumference, waist-hip ratio and conicity index in type 1 diabetes patients

**DOI:** 10.1186/1758-5996-7-S1-A86

**Published:** 2015-11-11

**Authors:** Camila Lemos Marques, Mileni Vanti Beretta, Raquel Eccel Prates, Filipe Valvassori Nascimento, Ciglea Nascimento, Jussara Carnevale de Almeida, Ticiana da Costa Rodrigues

**Affiliations:** 1 UFRGS, Porto Alegre, Brazil

## Background

Anthropometric measurements are used to evaluate the body adiposity distribution. Adiponectin is a protein synthesized by adipose tissue which has an inverse relationship with obesity and insulin resistance. Previous studies demonstrated that low plasma adiponectin levels are associated with type 2 diabetes mellitus and risk of cardiovascular disease. This study aims to evaluate the association between adiponectin levels and anthropometric measurements in patients with type 1 diabetes mellitus (T1D).

## Materials and methods

Cross-sectional study in outpatient adults with T1D in a hospital in southern Brazil, data collected from 2008 to 2013. The anthropometric measurements used were waist circumference (WC), waist-hip ratio (WHR), waist-to-height ratio (WHtR), body mass index (BMI), and conicity index (CI) and lipid accumulation product (LAP). Serum adiponectin was measured using a commercial ELISA kit (Invitrogen®, USA) and was analyzed in tertiles (tertile 1 ≤ 10.26 µg/mL, tertile 2 between 10.27 and 18.27 µg/mL and tertile 3 ≥ 18.28 µg/mL).

## Results

The 122 subjects studied (50.8% women) had mean age of 38.7±11.3 yrs. and median of adiponectin 13.1 µg/mL (8.8–22.9 µg/mL). Adiponectin was negatively correlated with WC (r=-0.19, p=0.04), WHR (r=-0.232, p=0.01) and CI (r=-0.18, p=0.04). Individuals of the first tertile of adiponectin (lowest levels) had higher values of WC, WHR and CI when compared with individuals of the others tertiles. After gamma regression analyses, adjusted for age, WC (Beta=-0.03; p<0.001), WHR (Beta=-4.86; p<0.001) and CI (Beta=-5.67; p<0.001) remained negatively associated with adiponectin. Variables like BMI, WHtR, LAP, total cholesterol, HDL-cholesterol, LDL-cholesterol, triglycerides, ultra sensitive C-reactive protein and insulin resistance were not associated with adiponectin levels in correlation analysis.

## Conclusions

Waist circumference, waist-hip ratio and conicity index were associated with low serum adiponectin levels in adults with T1D. The conicity index had a stronger association with adiponectin. As anthropometric measurements can be easily accessed through physical examination and might be indicative of cardiovascular risk in this population, this information should not be neglected by the health care professionals.

